# Atypical prediction error learning is associated with prodromal symptoms in individuals at clinical high risk for psychosis

**DOI:** 10.1038/s41537-022-00302-3

**Published:** 2022-11-25

**Authors:** Colleen E. Charlton, Jennifer R. Lepock, Daniel J. Hauke, Romina Mizrahi, Michael Kiang, Andreea O. Diaconescu

**Affiliations:** 1 Krembil Centre for Neuroinformatics, Centre for Addiction and Mental Health (CAMH), Toronto, ON Canada; 2 Centre for Addiction and Mental Health (CAMH), Toronto, ON Canada; 3 Institute of Medical Sciences, University of Toronto, Toronto, ON Canada; 4 Department of Psychiatry (UPK), University of Basel, Basel, Switzerland; 5 Department of Mathematics and Computer Science, University of Basel, Basel, Switzerland; 6 Douglas Research Centre, Montreal, QC Canada; 7 Department of Psychiatry, McGill University, Montreal, QC Canada; 8 Department of Psychiatry, University of Toronto, Toronto, ON Canada; 9 Department of Psychology, University of Toronto, Toronto, ON Canada

**Keywords:** Psychosis, Biomarkers

## Abstract

Reductions in the auditory mismatch negativity (MMN) have been well-demonstrated in schizophrenia rendering it a promising biomarker for understanding the emergence of psychosis. According to the predictive coding theory of psychosis, MMN impairments may reflect disturbances in hierarchical information processing driven by maladaptive precision-weighted prediction errors (pwPEs) and enhanced belief updating. We applied a hierarchical Bayesian model of learning to single-trial EEG data from an auditory oddball paradigm in 31 help-seeking antipsychotic-naive high-risk individuals and 23 healthy controls to understand the computational mechanisms underlying the auditory MMN. We found that low-level sensory and high-level volatility pwPE expression correlated with EEG amplitudes, coinciding with the timing of the MMN. Furthermore, we found that prodromal positive symptom severity was associated with increased expression of sensory pwPEs and higher-level belief uncertainty. Our findings provide support for the role of pwPEs in auditory MMN generation, and suggest that increased sensory pwPEs driven by changes in belief uncertainty may render the environment seemingly unpredictable. This may predispose high-risk individuals to delusion-like ideation to explain this experience. These results highlight the value of computational models for understanding the pathophysiological mechanisms of psychosis.

## Introduction

Schizophrenia is one of the most disabling mental disorders as it is associated with cognitive impairment, poor long-term social and occupational outcomes, and affects over 20 million people worldwide^1^. Recently, major clinical and research efforts have turned towards early intervention, identifying individuals at clinical high-risk (CHR) for psychosis, with the goal of preventing or postponing psychosis onset. Among CHR individuals, rates of transition to a psychotic disorder following clinical presentation are estimated to be 15% after 1 year, increasing to 25% after 3 years^2^. In this population, additional nonclinical measures are needed to clarify the pathophysiological mechanisms of psychosis^3^.

One such measure, termed the auditory mismatch negativity (MMN), has been described as a reproducible, reliable biomarker for understanding psychosis^4^. The auditory MMN is an event-related potential (ERP) involuntarily elicited in response to an infrequent sound stimulus that deviates from a repeated sequence of regular sound stimuli, e.g. in duration or frequency. The MMN serves as an index of surprise during sensory learning and deficits in the MMN amplitude have been shown to correlate with the level of function in chronic^4^ and first-episode schizophrenia^5^, and CHR individuals^6^. However, studies of the auditory mismatch response in CHR patients are variable, with some^7–12^, but not all^13–16^ previous studies reporting attenuated MMN amplitudes, and MMN abnormalities were found to be greatest in CHR participants who later convert to psychosis^17^. Recent pharmacological studies in healthy human controls reported significant MMN attenuation following the administration of ketamine, an *N*-methyl-d-aspartate (NMDA) antagonist^18^. Ketamine has previously been employed as an experimental model of psychosis^19,20^, and taken together, these results suggest that impairment in NMDA receptor function may contribute to MMN attenuation and symptoms of psychosis.

Computationally, the MMN has been interpreted from a predictive coding perspective as a prediction error (PE) signal that arises from a failure to anticipate an incoming stimulus^21–25^. In predictive coding and related theories of hierarchical Bayesian inference^26,27^, each level of a cortical hierarchy provides predictions about the state of the level below and evaluates the discrepancy with actual inputs from that level (i.e. PEs) to update predictions. Importantly, this updating process also depends on the relative weight or precision of the error signal (or its inverse, uncertainty), where the key aspect is the relative precision assigned to information from lower (sensory input) compared to higher levels (or top-down prior predictions).

From a hierarchical Bayesian perspective, the auditory MMN can be viewed as representing statistical learning about environmental regularities through sequential precision-weighted PE (pwPE) updates generated at multiple levels of the auditory hierarchy. Disturbances in this hierarchical information processing have been proposed to underlie psychotic symptoms^28,29^.

In recent years, the application of mathematical models to behavioural, electrophysiological, and neuroimaging data has allowed for the quantification of PE signals and a mechanistic understanding of psychotic symptoms as disruptions in hierarchical Bayesian inference^30,31^. Under this framework, psychotic symptoms are thought to originate from the misattribution of salience to irrelevant sensory stimuli, driving erroneous model updates and culminating in maladaptive predictions and unfounded beliefs (i.e. delusions)^28,32–34^. In generic hierarchical Bayesian formulations—such as the hierarchical Gaussian filter (HGF)—a ratio of precisions controls the influence of PEs on belief updating. In this theoretical framework, we place a greater emphasis on PEs when lower-level (sensory) inputs are more precise relative to higher-level predictions^35,36^. Based on this hierarchical Bayesian framework, two possible mechanisms underlying the emergence of psychotic symptoms have been proposed: first, a failure to attenuate lower-level sensory precision renders stimuli persistently surprising and gives rise to overly large learning rates, and thus a misattribution of salience to irrelevant stimuli^37,38^. Alternatively, a reduction in the precision of higher-level beliefs—i.e. increased informational uncertainty—may render the environment seemingly unpredictable, leading to an over-reliance on, and amplification of, low-level pwPE updates^28,31,39^. Notably, these explanations may co-exist, as they are not mutually exclusive. Both mechanisms result in aberrantly strong incoming PEs that outweigh prior predictions. These unexplained error signals may lead to a brittle or uncertain model of the world, thus predisposing an individual to the adoption of extraordinary unfounded higher-order beliefs culminating in delusions^29,31^.

In this paper, we conduct a computational, single-trial analysis of a previously published auditory MMN dataset in help-seeking antipsychotic-naïve CHR individuals and healthy controls (HC)^40,41^. The aim is to investigate the extent to which the trial-by-trial expression of hierarchically-related pwPEs underlies auditory MMN generation. Consistent with previous studies, we hypothesised that the MMN represents hierarchical PE learning^25,42,43^. Furthermore, based on predictive coding and hierarchical Bayesian theories of psychosis^29^, we hypothesise that the attribution of “aberrant salience” to objectively uninformative or neutral events^34^ is reflected in increased expression of low-level, sensory pwPEs. There has been recent empirical support for this notion in CHR populations where increased expression of sensory pwPEs in dorsolateral prefrontal cortex was associated with greater overall symptom severity in CHR individuals^39^.

## Results

**Fig. 1:**
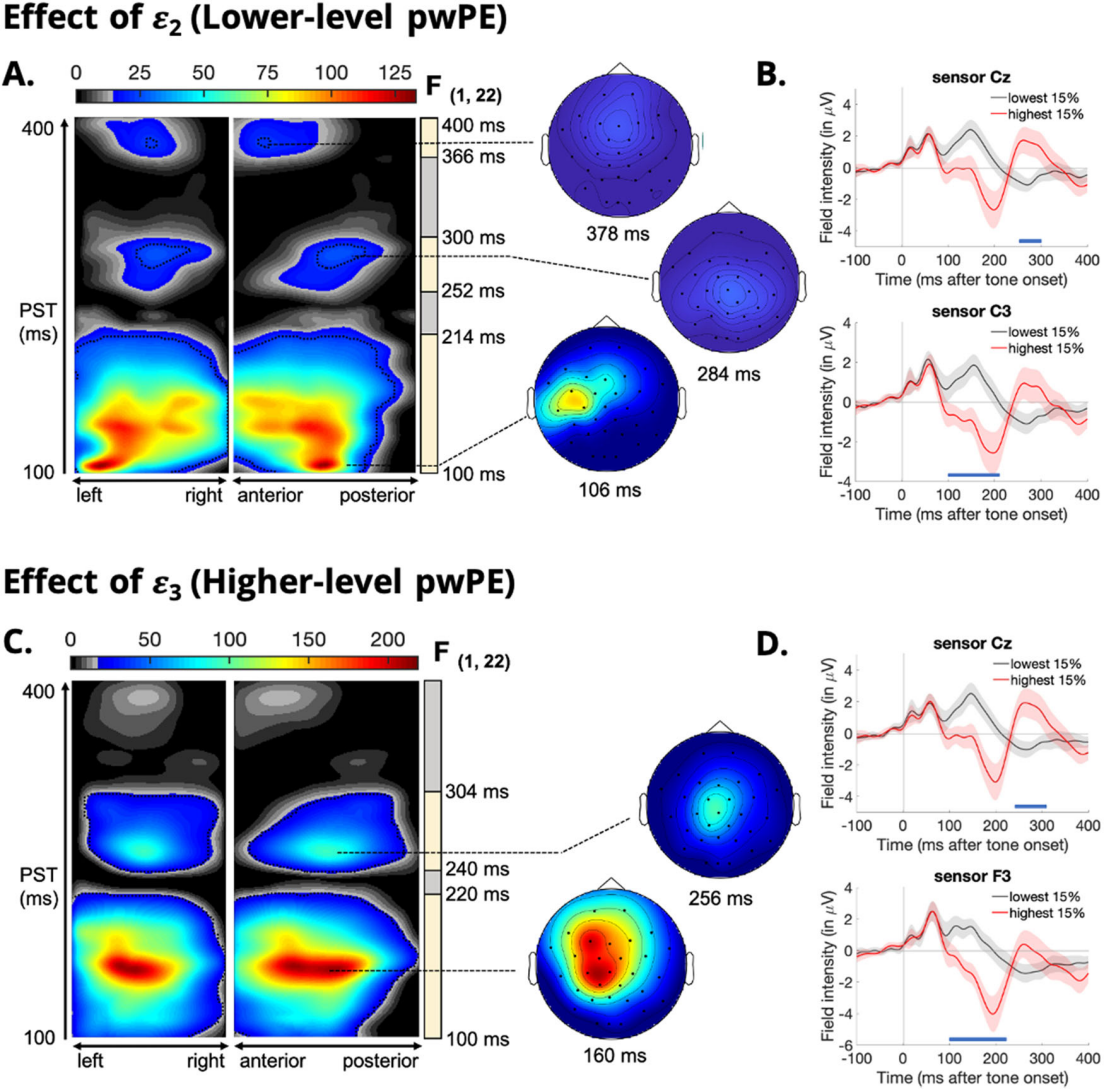
Expression of sensory (low-level) and volatility (high-level) precision-weighted prediction errors (pwPEs) in the control group. **A** Maximum intensity projections of the significant clusters over left to right and anterior to posterior scalp locations (left) of the F-statistic for low-level pwPEs (*ε*_2_). Significant cluster-level effects (*p* < 0.05, whole-volume family wise error (FWE) corrected at the cluster level with a cluster-defining threshold of *p* < 0.001) are shown using a jet colour-map and significant peak-level effects (*p* < 0.05, whole-volume FWE-corrected at the peak level) are marked by black contours. Coloured area highlights *f*-values that exceed the cluster-defining threshold of *p* < 0.001, uncorrected. Time windows of the significant effects (earliest to latest significant timepoints) are shown by yellow bars on the right of the F-map. The scalp maps (right) show the peak effect (global maximum) of the given cluster with an F-map at the indicated peristimulus time, across a 2D representation of the sensor layout. Note that the global peak effect is not always expressed at a specific channel location. Significant correlations with the low-level pwPE *ε*_2_ peaked at 106 ms in temporal central channels (sensor C3), at 284 ms in central channels (sensor Cz) and at 378 ms in frontal central channels. **B** Event-related potential waveforms averaged across the 15% highest and the 15% lowest pwPE values at electrodes within significant clusters. **C**, **D** Significant correlations with the high-level pwPEs *ε*_3_ peaked at 160 ms in frontal channels (sensor F3) and at 256 ms in central channels (sensor Cz).

**Fig. 2:**
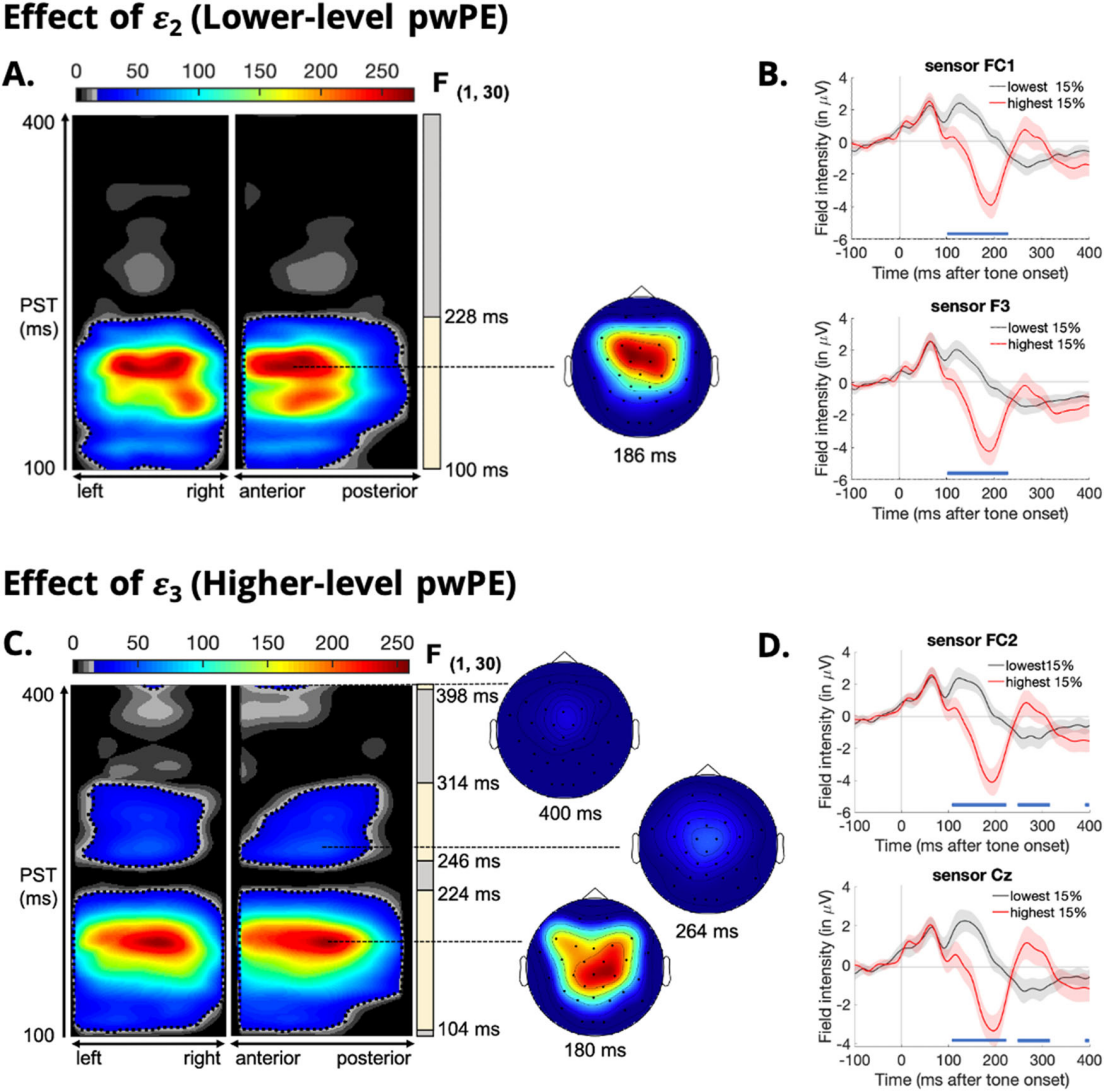
Expression of sensory (low-level) and volatility (high-level) precision-weighted prediction errors (pwPEs) in clinically high-risk group. **A** Maximum intensity projections of the significant clusters over left to right and anterior to posterior scalp locations (left) of the F-statistic for low-level pwPEs (*ε*_2_). Significant cluster-level effects (*p* < 0.05, whole-volume family wise error (FWE) corrected at the cluster level with a cluster-defining threshold of *p* < 0.001) are shown using a jet colour-map and significant peak-level effects (*p* < 0.05, whole-volume FWE-corrected at the peak level) are marked by black contours. Coloured area highlights *f*-values that exceed the cluster-defining threshold of *p* < 0.001, uncorrected. Time windows of significant cluster-level effects (earliest to latest significant timepoints) are illustrated by yellow bars on the right of the F-map. The scalp maps (right) show the peak effect of the given cluster with an F-map at the indicated peristimulus time, across a 2D representation of the sensor layout. Note that the global peak effect is not always expressed at a specific channel location. Significant correlations with the low-level pwPE *ε*_2_ peaked at 186 ms in frontocentral channels (sensor F3 and FC1). **B** Event-related potential waveforms averaged across the 15% highest and the 15% lowest pwPE values at electrodes within the significant clusters. **C**, **D** Significant correlations with the high-level pwPE *ε*_3_ peaked at 180 ms and 264 ms in central channels (sensor Cz), and at 400 ms in frontocentral channels (sensor FC2).

To identify the EEG correlates of pwPEs, we used a general linear model (GLM) with these computational variables to explain the observed trial-by-trial ERP responses, over channels and peri stimulus time (PST) with respect to tone onset. Significant correlations between model-based pwPE trajectories and single-trial ERPs was demonstrated in both groups with several significant scalp x time clusters. Significant effects are summarised in Figs. 1 and 2 using maximum intensity projections of the significant clusters over left to right scalp locations, retaining the anterior-posterior and PST dimensions in the plots. In both groups, the expression of hierarchical pwPEs ($\varepsilon _2,\varepsilon _3$) was not significantly correlated with sex or age.

### Expression of pwPEs in healthy controls

In HC, there were significant correlations between sensory (low-level) pwPEs (*ε*_2_) and EEG amplitudes between 100 and 214 ms post-stimulus, corresponding to a negative potential and peaking at 106 ms in temporal central channels (peak, *F*_(1,22)_ = 133.1; *p* = 6.1e-7; Fig. 1A). In this cluster, higher low-level pwPE values, or more surprising events, correlated with more negative EEG amplitudes. The timing of this early cluster coincided with the timing of the auditory MMN. Further effects of low-level pwPEs were observed in central channels between 252 and 300 ms post-stimulus corresponding to a positive potential and peaking at 284 ms (peak, *F*_(1,22)_ = 28.7; *p* = 0.0207; Fig. 1A). The timing of this effect coincided with the positive-P3a component, with larger low-level pwPEs correlating with an increased central positivity. Finally, a late cluster occurred between 366 and 400 ms post-stimulus, corresponding to a negative potential and peaking at 378 ms in frontal channels (peak, *F*_(1,22)_ = 24.4; *p* = 0.0458; Fig. 1A). The timing of this late cluster may continue past our analysis time window of 100 to 400 ms post-stimulus and may be indicative of the reorienting negativity (RON) ERP component^44^. The RON component typically peaks around 400 ms, signifying attentional reorientation and has been shown to be attenuated in first-episode psychosis and chronic-schizophrenia patients^45,46^.

Further correlations between single-trial ERPs and volatility (high-level) pwPEs (*ε*_3_) in the HC group resulted in multiple significant activation foci. The earliest significant cluster occurred between 100 and 220 ms post-stimulus, peaking at 160 ms in frontocentral channels (peak, *F*_(1,22)_ = 218.5; *p* = 7.1e-09; Fig. 1C). A later cluster occurred between 240 and 304 ms post-stimulus, peaking at 256 ms in central channels (peak, *F*_(1,22)_ = 97.8; *p* = 5.2e-06; Fig. 1C). Again, the timing of the high-level pwPE effects coincided with the timing of the MMN and P3a components, respectively. Ergo the MMN component, and to a lesser extent the P3a, may capture differences in one’s beliefs about environmental volatility.

### Expression of sensory (low-level) and volatility (high-level) pwPEs in the clinically high-risk group

In the CHR group, there were significant trial-wise correlations between low-level pwPEs (*ε*_2_) and EEG amplitudes between 100 and 228 ms post-stimulus, corresponding to a negative potential and peaking at 186 ms in frontal channels (peak, *F*_(1,30)_ = 275.8; *p* = 2.4e-12; Fig. 2A). Once again, this cluster coincides with the timing of the auditory MMN.

Three significant activation clusters occurred for trial-wise correlations with high-level pwPEs (*ε*_3_). The first cluster appeared between 104 and 224 ms post-stimulus, peaking at 180 ms in central channels (peak, *F*_(1,30)_ = 258.8; *p* = 4.4e-12; Fig. 2C), a second cluster occurred between 246 and 314 ms post-stimulus, peaking at 262 ms in central channels (peak, *F*_(1,30)_ = 56.1; *p* = 4.9e-05; Fig. 2C), and a third cluster occurred between 398 and 400 ms post-stimulus, peaking at 400 ms in frontocentral channels (peak, *F*_(1,30)_ = 23.9; *p* = 0.0212; Fig. 2C). Similar to low-level pwPE expression in HCs, the three significant clusters associated with high-level pwPE expression mirrored the series of negative-positive-negative waveforms reflective of the MMN/P3a/RON component complex. This complex provides a neurophysiological index of involuntary attention controls following a deviant stimuli^46^, which may be reflected by hierarchical PE updates.

### Group differences in sensory learning

Details of test statistics for HC and CHR groups are given in Tables S2 and S3, respectively. No significant group differences were found in the expression of *ε*_2_, the low-level pwPE about stimulus probability, or in the effects of *ε*_3_, the high-level pwPE about environmental volatility. Additionally, the expression of the hierarchically-coupled uncertainty trajectories ($\sigma _1,\sigma _2,\sigma _3$), unweighted PEs ($\delta _1,\delta _2$) and binary tones, did not differ between groups. Note that for $\sigma _3$, there were no surviving voxels in either group. The absence of group differences in our study was not surprising. Our dataset was primarily aggregated from Lepock and colleagues’^41^ who found no group differences in classical MMN amplitudes. See Fig. S1 for average ERPs of the classic MMN response (standard – deviant tones), and pwPE difference waveforms (15% highest − 15% lowest *ε*_2_ and *ε*_3_ trials, respectively) at electrodes within significant clusters close to the peak effect for both groups.

### Correlations between model parameters and clinical variables

Of the 31 CHR, three CHR converted to psychosis during a 2-year follow-up period. Due to the low number of converters, we could not examine whether differences in parameter expression was related to a subsequent conversion to psychosis. Instead, we tested for correlations between the effects of the hierarchical pwPEs ($\varepsilon _2,\varepsilon _3$) and positive prodromal symptom severity (SIPS) at baseline.

We cannot exclude the possibility that the three CHR converters had an outsized effect on correlations between model parameters and positive symptom severity. We therefore additionally report the results of our high-risk correlation analysis after excluding these data sets.

#### Increased expression of low-level sensory prediction errors in the clinically high-risk group

**Fig. 3:**
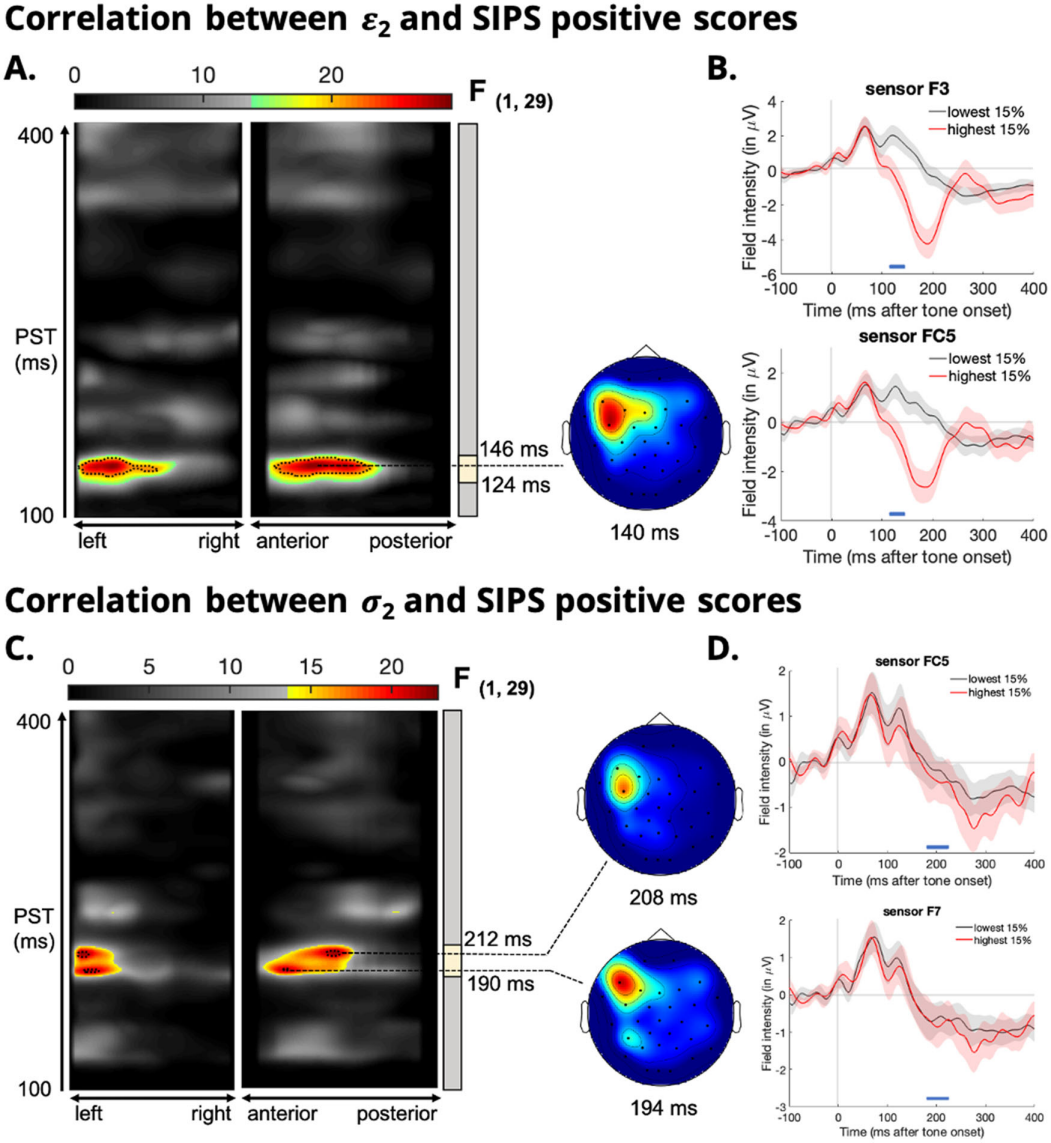
Significant positive correlations between prodromal positive symptom severity and both sensory (low-level) pwPEs and informational uncertainty in the clinically high-risk group. **A** Maximum intensity projections (MIP) of the significant clusters over left to right and anterior to posterior scalp locations (left) of the F-statistic for low-level PEs (*ε*_2_). Significant cluster-level effects (*p* < 0.05, whole-volume family wise error (FWE) corrected at the cluster level with a cluster-defining threshold of *p* < 0.001) are shown using a jet colour-map and significant peak-level effects (*p* < 0.05, whole-volume FWE-corrected at the peak level with a cluster-defining threshold of *p* < 0.05) are marked by black contours. Coloured area highlights *t*-values that exceed the cluster-defining threshold of *p* < 0.001, uncorrected. Time windows of significant cluster-level effects (earliest to latest significant timepoints) are marked by yellow bars on the right of the T-map. The scalp maps (right) show the peak effect of the given cluster at the indicated peristimulus time, across a 2D representation of the sensor layout. Note that the global peak effect is not always expressed at a channel location. Significant correlations between *ε*_2_ and prodromal positive symptom scores peaked at 140 ms in frontocentral channels (sensors FC5 and F3). **B** ERP waveforms averaged across the 15% highest and the 15% lowest PE values at electrodes within the significant clusters. **C**, **D** Significant correlations with informational uncertainty *σ*_2_ peaked at 194 ms in frontal channels (sensor F7) and 208 ms in frontocentral channels (sensor FC5).

We found a significant positive correlation between *ε*_2_ and SIPS positive-symptom subscale total scores between 124 and 146 ms post-stimulus peaking at 140 ms in frontocentral electrodes (peak, *F*_(1,29)_ = 29.3; *p* = 0.009; Cohen’s f^2^ = 0.338; Fig. 3A). This result also held when excluding the three CHR who later converted to psychosis (now CHR = 28; peak, *F*_(1,26)_ = 26.4; *p* = 0.020; Cohen’s f^2^ = 0.340). Simply put, an increased expression of low-level pwPE values were associated with greater SIPS positive subscale total scores at baseline (i.e. greater psychosis symptom severity).

#### Increased expression of informational belief uncertainty in the clinically high-risk group

We additionally tested three possible mechanisms underlying the positive correlation between low-level sensory pwPE updates and positive prodromal symptom severity in the CHR group: aberrantly large sensory PE updates (*δ*_1_), aberrantly high precision of incoming PEs (i.e. irreducible uncertainty, *σ*_1_), or aberrantly low high-level belief precision (i.e. informational uncertainty, *σ*_2_), all of which could lead to an increase in belief updating (see Eq. (1)).

We observed that *σ*_2_ showed significant correlations with SIPS positive symptoms in the CHR group between 190 and 212 ms post-stimulus, peaking at 194 ms (peak, *F*_(1,29)_ = 21.9; *p* = 0.045; Cohen’s f^2^ = 0.227; Fig. 3C) in frontal channels and 208 ms (peak, *F*_(1,29)_ = 22.9; *p* = 0.037; Cohen’s f^2^ = 0.242; Fig. 3C) in frontocentral channels. However, this effect did not hold when excluding the three CHR converters (now CHR = 28; peak, *F*_(1,26)_ = 13.3; *p* = 0.341; Cohen’s f^2^ = 0.129). In other words, CHR individuals with greater prodromal positive symptom severity at baseline exhibited increased effects of informational uncertainty. No significant correlations were found between SIPS positive symptoms and sensory PEs (*δ*_1_) or irreducible uncertainty (*σ*_1_). For completeness, we also investigated the effect of environmental uncertainty (*σ*_3_) (c.f.^22^) but found no significant effects. These results also held when excluding CHR converters. See Supplementary Information for a description of the correlations between informational uncertainty and single-trial ERPs in the HC and CHR group.

## Discussion

In this study, we combined computational modelling with trial-wise analyses of ERP responses to examine atypical PE learning in CHR individuals compared to HCs. Current predictive coding theories of psychosis suggest that positive symptoms, such as hallucinations and delusions, emerge from the misattribution of salience to irrelevant sensory stimuli driving specious model updates and culminating in unsound beliefs^28,32,33^. Model updating in the auditory hierarchy has been proposed to underlie MMN generation, and our computational modelling approach allows for separation of the MMN component into two hierarchically-coupled PEs: low-level sensory PEs and high-level volatility PEs. In both HC and CHR groups, our model-based analysis revealed significant correlations between single-trial EEG activity and both low- and high-level pwPE trajectories that coincided with the timing of the MMN and P3a response (see Figs. 1, 2). These findings are consistent with previous studies that modelled single-trial MMN responses of visual oddball^42^ and auditory roving paradigms^43^, and taken together, these results offer compelling evidence for the role of hierarchical PEs during auditory perceptual inference and implicit, statistical learning.

We did not find any significant differences between the CHR and HC groups in low- or high-level pwPEs. Importantly, however, we found that within the CHR group, prodromal positive symptom severity was positively correlated with larger low-level sensory pwPE updates (Fig. 3A). We further examined the mechanisms underlying the expression of heightened low-level pwPEs and found that positive symptom severity was correlated with the expression of informational uncertainty (i.e. the inverse of belief precision), however this effect did not hold following the removal of CHR converters. Despite this lack of significance, we believe our findings still hold merit. Amongst the CHR group, the three converters displayed higher than average positive symptom severity (SIPS positive scores were 13, 16 and 22, respectively). Hence CHR individuals with the greatest positive symptom burden also had greater expression of informational uncertainty. This suggest that impairments in predictive processing, demonstrated by increased information uncertainty, may represent a core neurophysiological deficit already present in those at highest risk of developing psychosis and may highlight an important predictive mechanism in psychosis progression.

Specifically, these findings suggest that CHR individuals with a greater positive symptom burden exhibit increased uncertainty about their internal model of the causes of sensory inputs, thus rendering the environment seemingly more unpredictable. This may, in turn, further increase learning from incoming PEs, which may be perceived as aberrant salience of the underlying stimuli. Over time, this may lead to adoption of unusual, incorrect higher-order beliefs that may become resistant to disconfirmatory evidence (i.e. delusions), as explanations for these experiences. Our results are consistent with those of Hauke and colleagues^47^, who using the same computational approach, found increased expression of low-level pwPEs, peaking at 137 ms in central channels, in CHR individuals who later converted to psychosis. Furthermore, these findings are in line with those of Cole and colleagues^39^ wherein computational modelling was applied to CHR individuals’ behaviour during a probabilistic learning paradigm. Model-based fMRI analyses revealed that larger low-level pwPE effects in the dorsolateral prefrontal cortex correlated with lower global functioning.

Based on this computational approach, we suggest that the MMN is related to both low- and high-level pwPEs. Recently, a similar computational modelling method was used to investigate the effects of ketamine on the MMN^43^. By using a crossover, placebo-controlled design during an auditory roving paradigm, the authors replicated previous findings showing that ketamine significantly reduced the auditory MMN in HCs^43,48^, while also showing that ketamine reduced the expression of high-level (volatility) pwPEs^43^. These results provide further support for the dysconnectivity hypothesis, which proposes that abnormal neurotransmitter modulation of NMDA receptor mediated plasticity may give rise to psychosis symptoms^49^. Taken together, increased MMN attenuation throughout the illness course, may be due to progressive NMDA receptor dysfunction, and reflected computationally by reductions in the expression of high-level pwPE updating^47^. In comparison, increased low-level pwPE expression may be reflective of the early stages of psychosis, where individuals attribute increased salience to irrelevant stimuli giving rise to sensory PEs. In other words, our finding of correlations between low-level pwPEs and positive prodromal symptoms in CHR do not necessarily contradict previous findings of MMN reductions in CHR populations^7–12^.

In line with this hypothesis, we reproduced Lepock and colleagues’^41^ results that found no group differences in classical MMN amplitudes. Divergent findings have been reported with regard to reductions in the MMN amplitude in the CHR population. In a meta-analysis, Erickson, Ruffle and Gold^17^ found that the overall effect size of MMN amplitudes in CHR participants who converted to psychosis vs. controls was large (0.79) and that CHR participants who did not convert to psychosis had a nonsignificant effect size of 0.17, which was statistically indistinguishable from controls. Thus, within CHR groups, MMN abnormalities may be a sensitive marker of clinical progression. Because MMN impairments are greater among CHR individuals who later convert to psychosis and MMN deficits progressively worsen over illness progression^17^, the absence of group differences in our study was not surprising given the low number of converters. In addition, an advantage of our study is that we used a more graded measure of symptom severity (the SIPS Positive subscale score) than conversion to psychosis, and this measure could be more sensitive to relationships between disease progression and MMN parameters. For example, due to the low number of converters, we could not examine whether a more pronounced expression of low-level pwPEs was related to a subsequent conversion to psychosis. However, the correlation between positive prodromal symptom severity and low-level pwPE updates suggest that impairments in predictive processing may represent a core neurophysiological deficit already present in those at highest risk of developing psychosis.

Our findings linking pwPE signalling and symptom expression help elucidate the progressive pathophysiology of psychosis and model-based parameters may hold great promise as objective neurophysiological markers for predicting transition to psychosis. Nonetheless, several important limitations remain. In the present study, the observed tone contingencies remained stable over all trials. Hierarchically-related pwPEs are best disentangled when the stream of sensory input (i.e. tones) exhibit changing volatility, thus allowing higher-level volatility PEs to be disentangled from low-level outcome PEs. Given the stability of our task structure, strong correlations were found between low-level and high-level pwPE expression rendering it difficult to isolate the effects on learning about volatility. An additional limitation in our methodology was the assumption of a Bayes-optimal learner for all participants. The MMN paradigm is a passive task and did not require behavioural responses from participants, hence the HGF model could not be fit to behaviour. Consequently and in line with previous methods^42,43^, we fit an optimal Bayesian learner model to the tone sequence that each participant perceived to obtain belief trajectories reflecting adaptive learning. Additionally, since participants did not have to respond behaviourally to the task, model selection based on behaviour could not be performed. In the future, it would be valuable to apply the same computational modelling approach performed here, to data collected during an active oddball task e.g. ref. ^50^, and test it against other theoretical models of MMN generation. A third limitation is our modest sample size, which may have limited the power to identify group differences in PE expression. Longitudinal follow-up in larger samples is needed to determine whether model-based parameters are predictive of illness progression, including conversion to psychosis. Finally, given the correlational nature of our study, the results are therefore consistent with a relation between MMN indices of PEs to psychosis-like symptoms, but do not prove that this relation is causal.

In conclusion, our results highlight the role of hierarchically-related pwPEs in auditory MMN generation and suggest that positive prodromal symptom severity is associated with increased outcome-related learning (low-level pwPEs). Furthermore, we validated the use of mechanistic computational models for understanding the emergence of prodromal symptoms in psychosis.

## Methods and materials

### Participants

We included 31 CHR and 23 HC participants aggregated from two previous studies^40,41^. Note that 41 CHR individuals had originally been recruited but ten participants were excluded from further analysis for a variety of reasons including concurrent disorders (*n* = 2; epilepsy and alcohol use disorder), electrode failure (*n* = 1), failure to sufficiently correct for eye-blink artefacts during preprocessing of EEG data (*n* = 6) and missing SIPS negative data (*n* = 1). Groups were matched for age and pre-morbid IQ to help ensure that any group differences in MMN measures were likely to be related to the disease process and not to pre-existing intellectual differences^51–53^. CHR participants were help-seeking outpatients referred to the Focus on Youth Psychosis Prevention outpatient clinic at the Centre for Addiction and Mental Health (CAMH) in Toronto. HCs were recruited from the community through advertisements online, in newspapers and on bulletin boards. All participants gave written informed consent and the study was approved by the CAMH Research Ethics Board. CHR individuals met diagnostic criteria for a psychosis-risk syndrome using the Criteria of Psychosis-Risk States based on the Structured Interview for Psychosis-Risk Syndromes (SIPS)^54^. All CHR participants were of the attenuated psychosis syndrome subtype^55^ and had no history of current or lifetime DSM-IV-TR Axis I psychotic disorder, or mood disorder with psychotic features^56^, as determined via the Structured Clinical Interview for DSM-IV-TR^57^. Participants had no history of DSM-IV substance use or dependence in the last 6 months (except nicotine) and were antipsychotic-naive. At two-year follow-up, three CHR individuals had converted to psychosis (*n* = 10 CHR dropped out of the study before two-year follow-up). Table 1 shows group demographic, neuropsychological and clinical characteristics.

**Table 1:** Participant characteristics.

	Healthy Controls (*n* = 23)	CHR Participants (*n* = 31)
Age (years)	21.5 (2.8)	20.7 (2.4)
Sex	13 female, 10 male	10 female, 21 male
Handedness	20 right, 3 left	28 right, 3 left
Parental socioeconomic status^74^	53.1 (13.9)	53.1 (13.8)
Years of education^a^	15.1 (1.8)	14.0 (2.1)
National adult reading test^75^ estimated pre-morbid verbal IQ	110.3 (5.9)	110.1 (9.6)
Scale of psychosis-risk-symptoms, Based on the structured interview for psychosis-risk syndromes (SIPS^54^;)		
Positive scale total		10.5 (3.6)
Negative scale total		12.8 (4.9)
Disorganised scale total		5.6 (3.2)
General scale total		9.6 (4.0)

Demographic, neuropsychological and clinical characteristics of the study sample (means, and in parentheses standard deviation, given for continuous variables).

^a^Patients differed significantly from controls, *p* = 0.04.

### Experimental paradigm

Electrophysiological (EEG) was recorded during an auditory oddball paradigm based on previous established protocols^58–62^. Participants were presented with 1830 tones on average binaurally through foam-inserted earphones (Model ER-3C, Etymotic Research, Elk Grove Village, IL) with an interstimulus interval of 500 ms. All tones were 85-dB, 1-kHz and had a rise-fall time of 1-ms. Standard tones (50 ms) and deviant tones (100 ms) were presented in pseudorandom order in 90% and 10% of the trials, respectively. Meanwhile, participants watched a silent cartoon video to divert attention from the tones.

### EEG collection and preprocessing

A 32-electrode EEG cap (Ag/AgCl, actiCAP system, Brain Products) with an actiCHamp amplifier (Brain Products, Gilching, Germany) was used. The electrodes were embedded in the cap at sites approximately equally spaced across the scalp, positioned according to a modified International 10–20 System (Fp1-Fp2-F7-F3-Fz-F4-F8-FC5-FC1-FC2-FC6-T7-C3-Cz-C4-T8-TP9-CP5-CP1-CP2-CP6-TP10-P7-P3-Pz-P4-P8-PO9-O1-Oz-O2-PO10). Eye movement artefacts (blinks and eye movements) were monitored via electrodes on the supraorbital and infraorbital ridges and on the outer canthi of both eyes. Continuous EEG recordings were referenced online to electrode FCz, and continuously digitised at 500 Hz. Electrode impedance was kept below 25 kΩ. EEG data were pre-processed using SPM 12 (http://www.fil.ion.ucl.ac.uk/spm/; version 7771). Continuous EEG was bandpass-filtered using a Butterworth filter between 0.5 and 30 Hz. The eye-blink correction procedure was consistent with previous single-trial computational analyses of EEG data^43^. Eye-blinks were detected using a threshold routine on the vertical EOG channel and all trials overlapping with eye-blink events were rejected. Corrected data was epoched into 500 ms segments around tone onsets (−100 to 400 ms) and baseline-corrected using a −100 to 0 ms prestimulus baseline. Remaining artefactual trials were rejected if the signal exceeded 100 μV. Channels were rejected and interpolated for sensor-level statistics if more than 20% of trials in a given channel were artefactual.

Bad channels occurred for one HC (O1) and two CHR (channel F4 and channels FP1 and FP2, respectively) participants. The average number of artefact-free trials was 1423 (SD, 173) for HCs and 1359 (SD, 161) for CHR; the number of remaining trials did not differ significantly between groups (see Table S1). ERPs were analyzed by including all artefact-free trials.

### Computational model

**Fig. 4:**
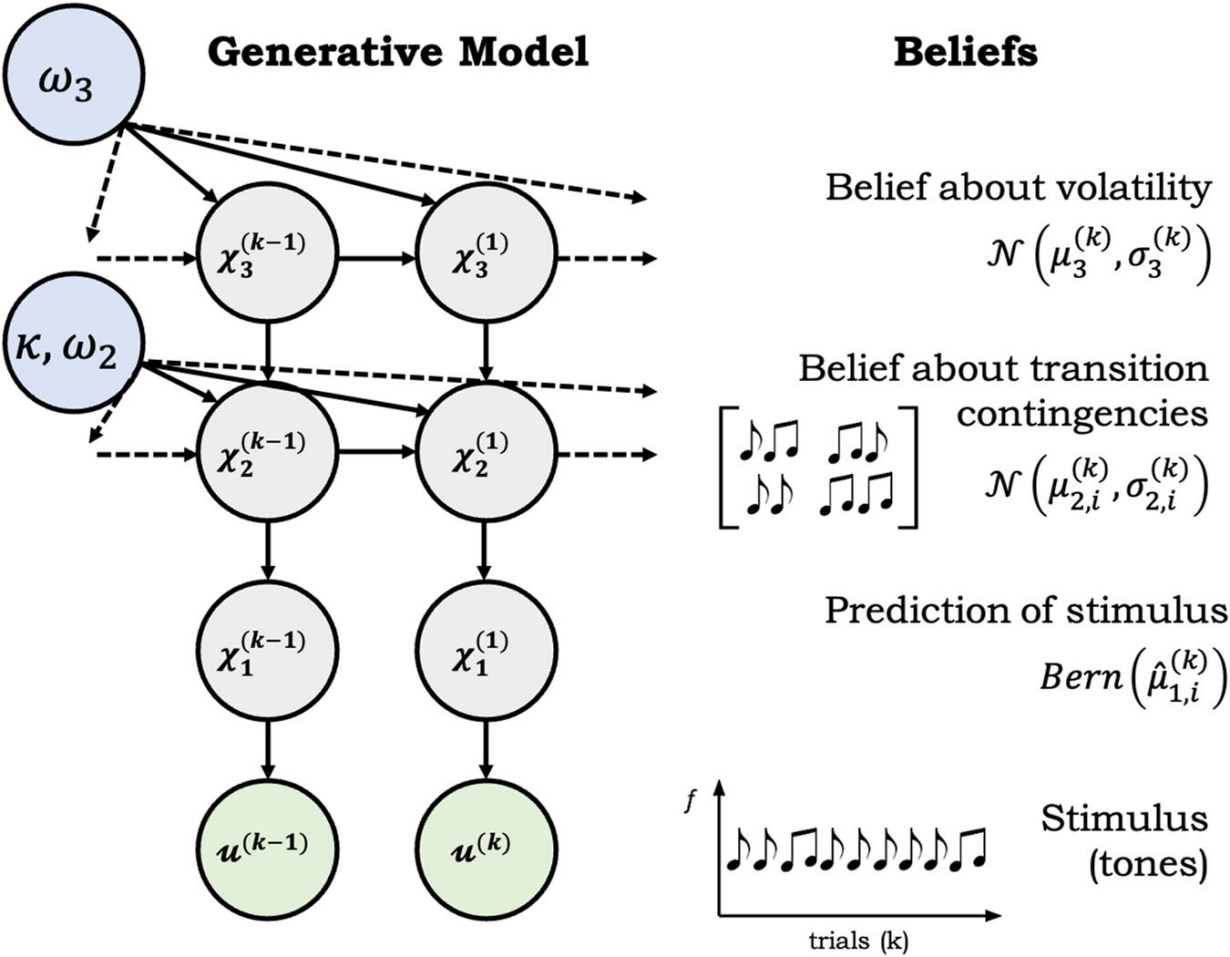
The three-level HGF binary perceptual model. An agent infers on multiple hidden states at time point k: stimulus probabilities (x_1_^(k)^), stimulus transition contingencies (x_2_^(k)^) and the phasic environmental volatility (x_3_^(k)^). Given new sensory input u^(k)^, an agent updates their beliefs at each hierarchical level using simple one-step update equations of the sufficient statistics, the mean *μ* and the variance *σ*.

To model perceptual inference during the auditory oddball task, we employed the hierarchical Gaussian filter (HGF), a generic hierarchical Bayesian model of learning, previously used for computational analyses of behaviour^63–67^ and in the context of modelling single-trial MMN responses of visual oddball^42^ and auditory roving paradigms^43^. The HGF captures subject-specific approximations to ideal hierarchical Bayesian inference and describes an individual’s learning under uncertainty. Since the MMN paradigm did not require behavioural responses from participants, we simulated the participants’ belief trajectories using Bayes-optimal parameters, which are defined as the parameters that result in the minimal overall surprise in response to a sequence of tone stimuli presented to the participant, in a given session^36^. In our current application, we assume the agent infers on hidden states in the world (*x*) from sensory inputs (*u*), which is reflected through belief updating across the HGF hierarchy (Fig. 4).

#### Inversion of the model: the update equations

The HGF update equations are derived by variational model inversion and provide approximate Bayes-optimal rules for trial-by-trial updating of an agent’s beliefs, given the agent’s particular set of parameter values^35^. “Belief” refers to a posterior probability distribution as described by its sufficient statistics, i.e. mean μ and variance σ (or its inverse, the precision π) under Gaussian assumptions.

On trial *k*, an observed trial outcome *u* indicates whether the tone was a “standard” tone (i.e. 50 ms duration) with *u*^(*k*)^ = 0 or a “deviant” (100 ms) with *u*^(*k*)^ = 1. The observed outcome *u*^(*k*)^ induces a sensory PE or $\delta _1^{\left( k \right)}$, which along with its precision *π*_2_^(*k*)^ can be referred to as the sensory pwPE ${\it{\epsilon }}_2^{(k)}$ and it is used to update $\mu _2^{\left( k \right)}$ or the belief about tone transition contingencies. This update, in turn, leads to a volatility PE or $\delta _2^{\left( k \right)}$, which along with its precision *π*_3_^(*k*)^ can be referred to as the volatility pwPE or ${\it{\epsilon }}_3^{(k)}$. This quantity is used to update $\mu _3^{\left( k \right)}$ or the belief about environmental volatility. This cascade of hierarchical belief updates can be summarised as follows:

1 $${\Delta}\mu _i^{\left( k \right)} \propto \frac{{\hat \pi _{i - 1}^{\left( k \right)}}}{{\pi _i^{\left( k \right)}}}\delta _{i - 1}^{\left( k \right)}$$

where, $\mu _i^{\left( k \right)}$ denotes the posterior mean of the belief on each trial *k* at level *i* of the hierarchy.

The PE from the level below ($\delta _{i - 1}^{\left( k \right)}$) is weighted by a ratio of precisions: the precision of the prediction about the level below (sensory precision) ($\hat \pi _{i - 1}^{\left( k \right)}$) and the precision of the belief at the current level in the hierarchy ($\pi _i^{\left( k \right)}$). The precision-ratio is updated with each trial and at each hierarchical level serving as a dynamic learning rate and enabling the flexibility required to adapt to evolving outcome probabilities and environmental volatility. See Supplementary Information for further explanation of model parameters.

### Single-trial EEG analysis

To test whether trial-by-trial pwPEs correlated with EEG amplitude fluctuations, single-trial EEG data were converted into three-dimensional volumes with two spatial dimensions (anterior to posterior and left to right directions of the scalp surface) and a temporal dimension (peristimulus time) for all 32 channels and timepoints in the 100–400 ms post-stimulus interval using a voxel size of 4.25 ms × 5.38 ms × 2 ms. Bad channels were linearly interpolated and images were smoothed using a Gaussian kernel (full-width at half maximum: 16 mm × 16 mm) to ensure that assumptions of random field theory were met^68,69^. The scalp × time 3D images were subjected to statistical analysis using the GLM analogous to fMRI analyses^70^ and applied to scalp EEG data using the SPM software^71^. At the single subject level, we pursued single-trial analyses where the computational model of behavioural responses allowed us to quantify learning trajectories, including pwPE trajectories (${\it{\epsilon }}_2^{(k)},{\it{\epsilon }}_3^{(k)}$). Additional GLMs were included to unpack the effects of pwPEs: the hierarchically-coupled uncertainty trajectories ($\sigma _1^{(k)},\sigma _2^{(k)},\sigma _3^{(k)}$), and the unweighted PEs ($\delta _1^{\left( k \right)},\delta _2^{\left( k \right)}$). A final GLM incorporating the tone sequence was included to assess trial-by-trial predictions about categorical change (standard vs. deviant). For each computational quantity, we tested the null hypothesis that the parameter estimates were zero at each sensor and peristimulus time (PST).

A random-effects group analysis was performed across all subjects in each group. At the group level, significant effects were inferred, from thresholded *F* statistical parametric maps (SPM) that were family wise error (FWE) corrected using random field theory at peak level (*p* < 0.05) and at the cluster level (*p* < 0.05) with a cluster-defining threshold of *p* < 0.001^72^. Group differences were assessed using a paired *t*-test. All reported results survived whole-volume FWE correction at the peak-level (*p* < 0.05). A measure of the effect size, Cohen’s f^2^^73^, was calculated from the partial eta-squared using the reported *F*-statistic and degrees of freedom. Finally, we evaluate the impact of sex and age on pwPE expression at the group level by performing an analysis of covariance (ANCOVA).

### Relationship with clinical variables

We investigated the association between pwPE representation during single-trial ERPs and clinical measures of the CHR state. We performed an ANCOVA at the group level testing the association between pwPE effects and SIPS positive symptoms.

## Supplementary information

Revised Supplementary - Marked Up

## Data Availability

All relevant data are available from the authors upon reasonable request.

## Code availability

The code for statistical analyses and generated figures is available upon request.
