# Supplementary material for: Atypical prediction error learning is associated with prodromal symptoms in individuals at clinical high risk for psychosis
Source: Schizophrenia (Heidelb). 2022 Nov 25;8(1):105. doi: 10.1038/s41537-022-00302-3 (PMC9700713; doi:10.1038/s41537-022-00302-3)
Supplement: Supplementary file 1 — Revised Supplementary - Marked Up [file 41537_2022_302_MOESM1_ESM.pdf]

Supplementary

Computational model parameters

Three participant-specific perceptual parameters, κ, ω_2 and ω_3 modulate individual learning and belief trajectories. Parameter κ determines the coupling strength between the second and the third level, while ω_2 is the tonic component of the log-volatility at the second level, which determines the speed of learning about stimulus transition contingencies. Parameter ω_3 refers to the variability of volatility over time (meta-volatility) and determines the speed of learning about environmental volatility.

In our context, however, the auditory oddball task is a passive paradigm simulated participants' belief trajectories using Bayes-optimal parameters, which resulted in slightly different parameter values for each participant because, although the probability schedule was consistent across participants, the exact order of the tones differed across participants. The priors over the parameters are summarized in **Table S4** and **Table S5** provides a summary of the HGF parameters from the optimal Bayesian model inversion.

Expression of informational uncertainty in the control and clinically high risk group

In both the HC and CHR group, there were significant trial-wise correlations between informational uncertainty (σ_2) and EEG amplitudes. In the HC group, a significant cluster occurred between 100 and 146 ms post-stimulus, peaking at 100 ms (peak, $F_{(1,22)} = 33.40$; $p = 0.0100$; **Figure S2A**) and 136 ms (peak, $F_{(1,22)} = 34.21$; $p = 0.0088$; **Figure S2A**) in frontal central channels. In the CHR group, a significant cluster occurred between 100 and 168 ms post-stimulus, peaking

at 100 ms (peak, $F_{(1,30)} = 22.47$; $p = 0.0358$; **Figure S2C**) in frontocentral channels and 122 ms (peak, $F_{(1,30)} = 39.72$; $p = 9.3e\text{-}4$; **Figure S2C**) in frontal channels.

Supplementary Figures and Tables

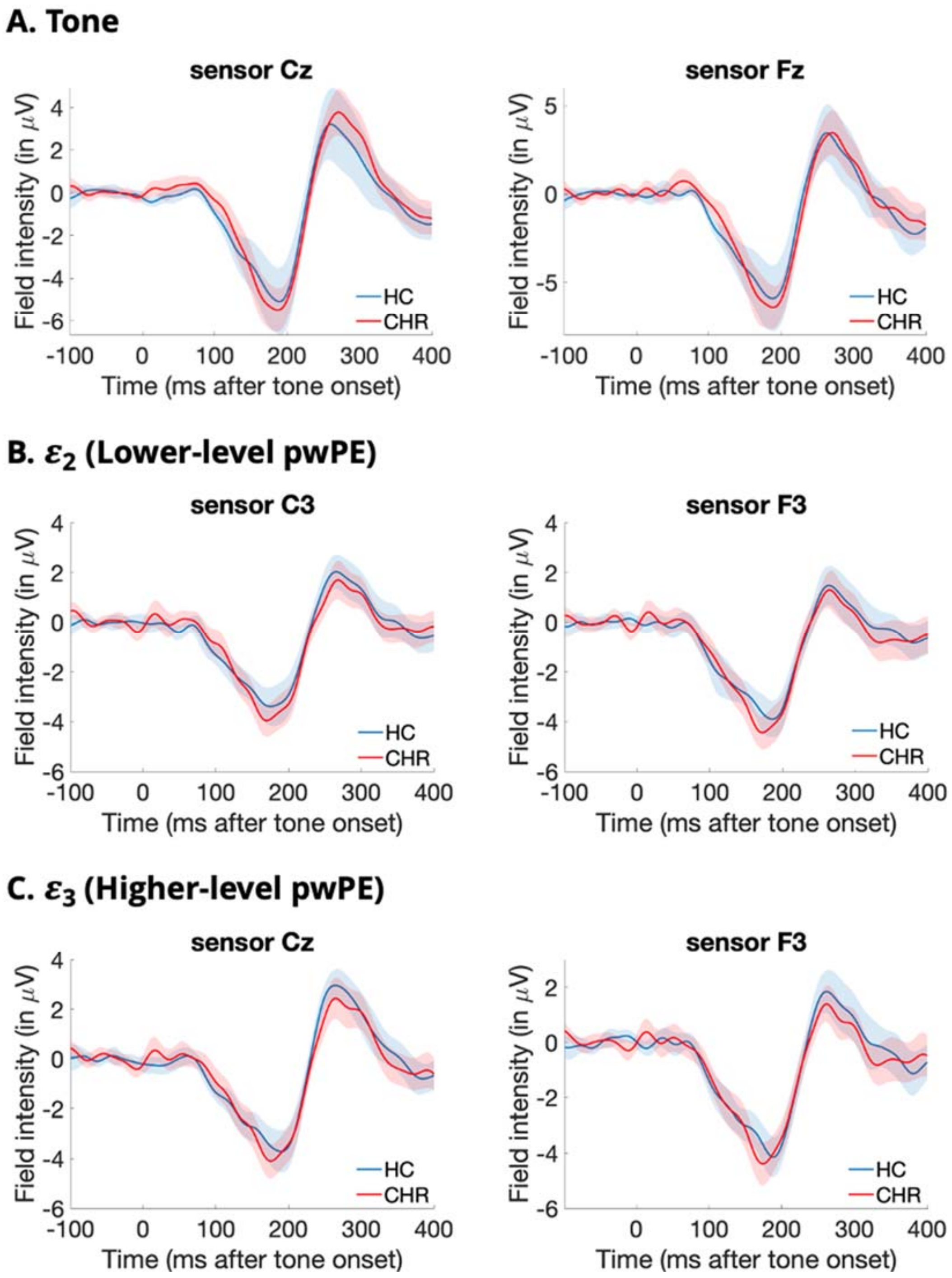

Figure S1: Average event-related potentials. *Average mismatch negativity waveform (standard - deviant tones)* ***(A)*** *and difference waveform (15% highest - 15% lowest* ε_2 *and* ε_3 *trials, respectively)* ***(B, C)*** *at electrodes within significant clusters close to the peak effect in the healthy control (HC) group and clinically high risk (CHR) groups.*

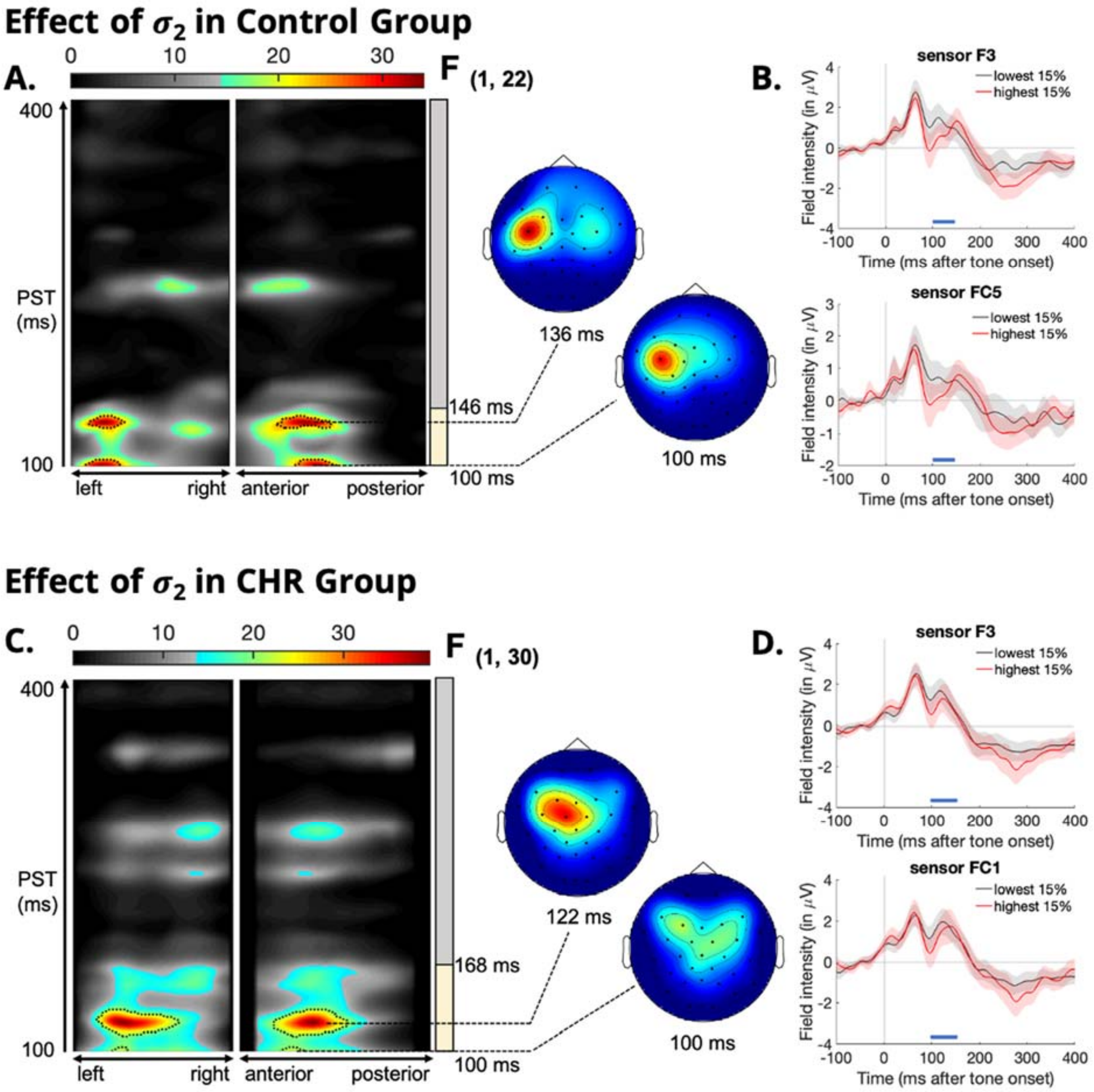

Figure S2: Expression of informational uncertainty in control and clinically high risk groups. ***(A)*** *Maximum intensity projections of the significant clusters over left to right and anterior to posterior scalp locations (left) of the F-statistic for the informational uncertainty (σ_2). Significant effects ($p < 0.05$, whole-volume family wise error (FWE) corrected at the cluster level with a cluster-defining threshold of $p < 0.001$) are shown using a jet colour-map and significant peak level F effects ($p < 0.05$, whole-volume FWE corrected at the peak level) are marked by black contours. Coloured area highlights f -values that exceed the cluster-defining threshold of $p < 0.001$, uncorrected. Time windows of significant cluster-level correlations (earliest to latest significant timepoints) are indicated by yellow bars on the right of the F map.*

The scalp maps (right) show the peak effect of the given cluster at the indicated peristimulus time, across a 2D representation of the sensor layout. Note that the global peak effect is not always expressed at a channel location. In the control group, significant effects of informational uncertainty σ_2 peaked at 100 ms and 136ms in frontocentral channel (sensors FC5 and F3). ***(B)*** *Event related potential waveforms averaged across the 15% highest and the 15% lowest uncertainty values at electrodes within significant clusters.* ***(C, D)*** *In the CHR group, significant effects of informational uncertainty σ_2 peaked at 100 ms and 122 ms in frontocentral channels (sensors FC1 and F3).*

Table S1: Trial statistics.

	Healthy Controls (n = 23)	Clinically High Risk (n = 31)	Group Differences
Eye-blink trials rejected	320 (103)	369 (111)	p=0.1064
Artefact trials rejected	93 (97)	102 (95)	p=0.7322

Mean and, in parenthesis, the standard deviation of the number of excluded and remaining trials per group.

Table S2: Test statistics for general linear model parameters in control group.

Computational parameter	Cluster size (voxels)	Cluster *p*-value (FWE-corrected)	Peak *p*-value (FWE-corrected)	F-value of peak voxel	Cohen's f^2	Peak location (mm, mm, ms)
ε_2	34798	**8.40E-10**	**6.07E-07**	133.10	2.794	-42, -14, 106
	3471	**0.0054**	**0.0207**	28.68	0.471	8, -25, 284
	2472	**0.0129**	**0.0458**	24.35	0.381	0, 40, 378
ε_3	36962	**1.41E-08**	**7.08E-09**	218.47	4.727	-21, 18, 160
	16272	**1.75E-05**	**5.24E-06**	97.80	1.998	0, -9, 256
	712	0.088	0.090	19.62	0.286	-4, 34, 386
δ_1	36958	**1.31E-08**	**4.64E-09**	229.47	4.977	-21, 18, 162
	16600	**1.47E-05**	**1.10E-05**	88.81	1.796	0, -14, 256
	2500	**0.018**	**0.038**	24.00	0.374	0, 34, 388
	4	0.258	0.255	14.59	0.189	47, -41, 330
δ_2	33025	**3.83E-08**	**2.72E-06**	106.79	2.200	-13, 72, 166
	6047	**0.0019**	**0.0042**	36.76	0.643	-4, 2, 266
	3720	**0.0077**	**0.0293**	25.49	0.405	-8, 18, 390
σ_1	124	0.236	0.239	16.47	0.224	-34, 13, 108
σ_2	1529	**0.0312**	**0.0088**	34.21	0.588	-34, 13, 136
			**0.0100**	33.40	0.571	-38, 2, 100
	288	0.168	0.118	19.91	0.291	38, 8, 130
	178	0.207	0.154	18.57	0.265	21, 34, 248
Tone	36716	**1.31E-8**	**5.68E-9**	224.34	4.860	-21, 18, 162
	16641	**1.39E-5**	**1.30E-5**	86.94	1.754	0, -9, 258
	3197	**0.011**	**0.025**	26.15	0.418	-4, 34, 388
	7	0.255	0.243	14.85	0.194	47, -41, 330

Significant values (p<0.05) are shown in bold.

Table S3: Test statistics for general linear model parameters in clinically high risk group.

Computational parameter	Cluster size (voxels)	Cluster *p*-value (FWE-corrected)	Peak *p*-value (FWE-corrected)	F-value of peak voxel	Cohen's f^2	Peak location (mm, mm, ms)
ε_2	34978	**1.28E-10**	**2.43E-12**	275.84	4.360	-17, 24, 186
	520	0.100	0.140	16.80	0.148	0, -14, 264
ε_3	33506	**2.08E-09**	**4.43E-12**	258.81	4.077	13, -19, 180
	12369	**2.00E-05**	**4.86E-05**	56.07	0.737	4, -9, 262
	1342	**0.0359**	**0.021**	23.93	0.245	8, 18, 400
	8	0.267	0.241	13.97	0.112	-26, 56, 326
δ_1	34256	**3.90E-09**	**6.12E-12**	250.47	3.939	17, -14, 180
	11489	**4.80E-05**	**6.94E-05**	53.35	0.694	0, -14, 260
	869	**0.0632**	**0.0273**	22.59	0.226	4, 24, 400
	23	0.239	0.163	15.35	0.129	-26, 56, 326
δ_2	30335	**1.37E-08**	**5.05E-11**	210.26	3.271	30. -9. 180
	5056	**0.0019**	**0.0068**	28.82	0.316	-4, -9, 258
	137	0.186	0.069	18.94	0.176	0, 40, 400
	26	0.245	0.134	16.28	0.141	-30, 56, 330
σ_1	284	0.157	0.144	17.51	0.157	0, 2, 120
σ_2	5993	**4.57E-04**	**9.30E-04**	39.72	0.481	-21, 8, 122
			**0.0358**	22.47	0.225	-26, 24, 100
	389	0.125	0.080	19.14	0.179	42, 2, 278
	39	0.271	0.241	14.64	0.121	38, 13, 242
	10	0.305	0.311	13.54	0.107	-8, 18, 276
Tone	33976	**4.15E-9**	**6.36E-12**	249.75	3.927	21, 14, 180
	11353	**5.06E-5**	**8.80E-5**	51.89	0.671	4, -14, 260
	909	0.060	**0.023**	23.43	4.360	4, 24, 400
	19	0.244	0.172	15.15	0.148	-26, 56, 326

Significant values (p<0.05) are shown in bold.

Table S4: Hierarchical Gaussian filter perceptual model parameters.

Parameter	Prior	
	Mean	Variance
κ	log(1)	0
ω_2	-3	4
ω_3	2	4
$\mu_2^{k=0}$	0	0.1
$\sigma_2^{k=0}$	0	0
$\mu_3^{k=0}$	1	1
$\sigma_3^{k=0}$	0	0

Summary of the prior mean and variance of the hierarchical Gaussian filter perceptual model parameters.

Table S5: Hierarchical Gaussian filter model inversion parameters.

	Healthy Controls (n = 23)	CHR Participants (n = 31)
ω_2	-1.402 (0.893)	-1.371 (0.705)
ω_3	4.743 (0.337)	4.788 (0.297)
$\sigma_2^{k=0}$	0.105 (0.007)	0. 109 (0.007)
$\sigma_3^{k=0}$	0.999 (0.003)	0.999 (0.002)

Summary of the hierarchical Gaussian filter parameters following model inversion for each group (means, and in parentheses standard deviation).
